# Efficacity and Safety of the Fluocinolone Acetonide Implant in Uveitic Macular Edema: A Real-Life Study from the French Uveitis Network

**DOI:** 10.3390/jpm14030245

**Published:** 2024-02-24

**Authors:** Matthieu Jabbour, Laurent Kodjikian, Alexandre Bourdin, Marie-Bénédicte Rougier, Yasmine Serrar, Michel Weber, Hélène Massé, Driss Mazhar, Sara Perez-Roustit, Christophe Chiquet, Marie Nöelle Delyfer, Bahram Bodaghi, Sara Touhami

**Affiliations:** 1 Department of Ophthalmology, Pitié Salpêtrière University Hospital, Sorbonne University, 75013 Paris, France; matthieujabbour.oph@gmail.com (M.J.);; 2 Department of Ophthalmology, Hôpital Universitaire de la Croix-Rousse, Hospices Civils de Lyon, University Claude Bernard Lyon 1, 69004 Lyon, France; laurent.kodjikian@chu-lyon.fr (L.K.);; 3 UMR5510 MATEIS, CNRS, INSA Lyon, University Claude Bernard Lyon 1, 69100 Villeurbanne, France; 4 CHU Bordeaux, Service d’Ophtalmologie, 33000 Bordeaux, Francemarie-noelle.delyfer@chu-bordeaux.fr (M.N.D.); 5 CHU Nantes, Service d’Ophtalmologie, Nantes University, 1 Place Alexis Ricordeau, 44093 Nantes, France; michel.weber@chu-nantes.fr (M.W.);; 6 Department of Ophthalmology, Grenoble Alpes University, 38043 Grenoble, France; 7 Laboratoire HP2, INSERM U1300, Grenoble Alpes University, 38000 Grenoble, France

**Keywords:** uveitic macular edema, dexamethasone, fluocinolone acetonide implant, chronic non-infectious uveitis, predictive factor, DRIL, hyperreflective foci

## Abstract

Purpose: To evaluate the safety and efficacy of the fluocinolone acetonide implant (FAi, Iluvien^®^ Horus pharma, Nice, France) in non-infectious uveitic macular edema (UME) and to approach the predictive factors of treatment response. Methods: This retrospective, multicenter real-life study included patients with chronic non-infectious UME who received intravitreal FAi after at least two dexamethasone implants (DEXi). Results: Twenty-six eyes from 22 patients (73.1% of females) were included. The mean age was 60.4 ± 16 years. The mean follow-up was 11.4 ± 2 months. The mean baseline best-corrected visual acuity (BCVA) was 0.43 ± 0.36 LogMAR, improving significantly after 1, 3, 6 and 12 months (all *p* < 0.05 vs. baseline). The mean baseline central macular thickness (CMT) was 429 ± 110 μm, improving significantly after 1, 3, 6 and 12 months (all *p* < 0.05 vs. baseline). Five eyes (19.2%) developed ocular hypertension during the follow-up, requiring initiation or strengthening of intraocular pressure lowering medication. The majority of eyes (77%) did not require any rescue DEXi during the available 12-month follow-up. The resolution of UME after DEXi seemed to predict the anatomical response after FAi. The baseline presence of a disorganization of the inner retinal layers (DRIL) and hyperreflective foci (HRF) were both associated with a higher likelihood of requiring rescue DEXi injections. Conclusion: FAi implantation led to a significant BCVA and CMT improvement with a good safety profile over the 12-month follow-up. Predictive factors of treatment outcomes seem to include the anatomical response to DEXi and the presence of DRIL and HRF at baseline.

## 1. Introduction

Uveitic macular edema (UME) results from intra and extracellular fluid accumulation within the macular region, due to inflammatory and ischemic signals that both induce a disruption of the blood–retinal barrier. It is one of the major causes of vision loss among patients with uveitis, resulting in a real societal burden [1,2]. When not treated promptly and adequately, UME can result in blindness. Today, many treatment options are available and include systemic steroids, immunosuppressive treatments with steroid sparing characteristics, systemic biotherapies and local treatments including mainly intravitreal dexamethasone (DEXi; Ozurdex^®^; Abbvie, Chicago, IL, USA) and fluocinolone acetonide (FA) implants (FAi). Systemic corticosteroids are highly efficient but cause metabolic and organ-specific complications that are poorly tolerated in the long term [3,4,5,6]. The advent of corticosteroid-sparing agents and biotherapies has dramatically decreased the treatment burden of corticosteroids; however, these drugs can come with their own complications. They can also be contraindicated in specific situations, urging researchers to propose local alternatives. DEXi has been used for more than a decade in macular edema due to diabetes, central retinal vein and uveitis [7,8,9,10]. Randomized controlled trials showed the efficacy of DEXi in uveitis, leading to a 6-fold higher chance of visual improvement when compared to the sham treatment. However, the outcomes of DEXi are not always favorable with up to 50% of UME patients being unresponsive or partly responsive. In addition, the durability of DEXi does not exceed four to six months [9,11,12,13,14,15], implying the recourse to iterative invasive intravitreal procedures. In this vein, sustained release implants offer an interesting alternative, allowing a longer-term quiescence. FA implants were designed in the early 2000s and were initially used in the form of surgically implanted devices (Retisert^®^ Bausch & Lomb Incorporated, Rochester, NY, USA) with a theoretical drug-release duration of 3 years [16,17]. However, the invasiveness of the surgical implantation and the high FA dosage (0.59 mg) were associated with local morbidity including the development of cataracts and severe glaucoma cases. The intravitreal FA implant from Alimera Sciences Inc. (Iluvien^®^, Alpharetta, GA, USA) consists in a non-biodegradable polymer with a length of 3.5 mm and a diameter of 0.37 mm, inserted through a 25 G intravitreal injection. It releases an FA dose of 0.2 μg/day, with a lower total dose of 0.19 mg delivered over a maximum time of 36 months, reducing the rate of incident cataracts and glaucoma cases [18,19] (p. 201). The efficacy of FAi in non-infectious uveitis of the posterior segment including UME (example in Supplemental Figure S1), was shown in a multicenter randomized trial including over 100 participants with a follow-up of 36 months [18,20]. This granted the fluocinolone acetonide implant both FDA and EMA approval in 2016 and 2018, respectively. Ever since, multiple real-life studies, mainly in a single-center setting, have confirmed its efficacy. The purpose of this work was not only to provide our real-life multicentric experience of FAi but also to try to approach the factors predicting the response to this treatment in patients with UME.

## 2. Methods

This was a retrospective, real-world, multicenter (Pitié Salpétrière Hospital Paris, Croix-Rousse Hospital Lyon, University Hospital of Bordeaux, Nantes University Hospital, Grenoble University Hospital) study involving consecutive patients with chronic noninfectious UME who were treated with FAi between September 2021 and October 2022.

The inclusion criteria were age > 18 years, non-infectious non-anterior uveitis associated with UME and having necessitated at least two DEXi prior to the treatment with FAi. The exclusion criteria were the following: macular edema due to another cause (diabetes, central retinal vein occlusion, post-surgical macular edema, etc.), severe glaucoma or uncontrolled ocular hypertension needing more than 2 antiglaucoma drops. The use of systemic corticosteroids and/or immunosuppressants and/or biologics was not an exclusion criterion. 

The study parameters were collected at baseline (i.e., before any injection); 2 months after the latest DEXi; and 1, 3, 6, and 12 months after FAi. The collected parameters included the best-corrected visual acuity (BCVA) measured on a decimal scale converted to LogMAR (logarithm of the minimum angle of resolution), intraocular pressure measured with a Goldman applanation tonometer, the presence or absence of intraocular inflammation features on slit-lamp examination according to the SUN (standardization of uveitis nomenclature) definitions [21], a measure of the central macular thickness (CMT) assessed by spectral domain optical coherence tomography (SD-OCT) (Spectralis, Heidelberg Engineering, Heidelberg, Germany) and UME characteristics: intraretinal cysts, subretinal detachment (SRD), the presence or absence of a disorganization of the inner retinal layers (DRIL) and the presence or absence of outer retina disruptions or hyper reflective foci (HRF) as seen on SD-OCT. 

Statistical analyses were performed using Stata (version 17, StataCorp, College Station, TX, USA). Continuous variables were expressed as the mean ± standard deviation (SD) and compared using the Wilcoxon matched-pairs signed-rank test, unpaired test or single-way ANOVA depending of the number of variables. Categorical data were expressed as proportions and were compared using Fisher’s exact test. GraphPad Prism 10.0.2 (GraphPad Software) was used for data analysis and graphic representation.

## 3. Results

### 3.1. Patients’ Characteristics at Baseline

Patient characteristics at baseline (before FAi) are presented in Table 1. Briefly, twenty-six eyes from 22 patients (16 females, 73.1%) of mean age 60.4 ± 15.8 years were included. The mean follow-up duration was 11.4 ± 2.0 months. Fifty-four percent of eyes had panuveitis (*n* = 14). The etiology of uveitis was the following: idiopathic (*n* = 15, 57.7%), sarcoidosis (*n* = 5, 19.2%), Vogt–Koyanagi–Harada (*n* = 2, 7.7%), autoimmune (*n* = 2, 7.7%), HLA-B27+ (*n* = 1, 3.8%) and immune restoration syndrome (*n* = 1, 3.8%). Regarding the characteristics of UME, HRF were present in 53.8% (*n* = 14) of eyes, SRF in 38.5% (*n* = 10) of eyes, and DRIL in 30.8% of cases (*n* = 8) at baseline. Twenty-five eyes (96.2%) were pseudophakic initially and six eyes (23.1%) were receiving anti-glaucoma drops (monotherapy: *n* = 3 (11.5%), dual therapy: *n* = 3 (11.5%)). The mean number of DEXi received prior to the first FAi was 8.5 ± 6.1, with a majority of eyes (*n* = 11, 42.3%) having received between 5 and 10 DEXi. The mean time between the latest DEXi and the first FAi was 5.0 ± 7.9 months (range: 0–29). Fifty-nine percent of patients (*n* = 13) were under systemic treatment with either corticosteroids (*n* = 3, 13.6%), immunosuppressive therapy (*n* = 5, 22.7%) or a combination of both (*n* = 5, 22.7%) before FAi.

### 3.2. Functional and Anatomical Outcomes of FAi

**Figure 1:**
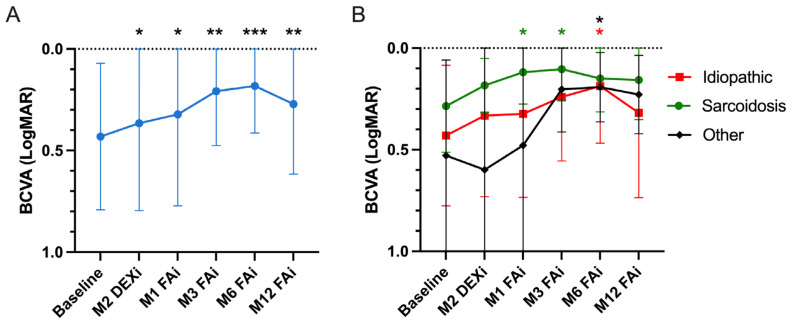
Evolution of the best corrected visual acuity (BCVA). (**A**) Whole cohort. (**B**) BCVA evolution according to uveitis etiology. “Baseline” refers to the pre-dexamethasone implant (DEXi) period. M: month, FAi: Fluocinolone acetonide implant. “Other” includes the following uveitis etiologies: Vogt–Koyanagi–Harada, autoimmune retinitis, HLA-B27+ and immune restauration syndrome. The graph represents the mean values with the corresponding standard deviation. Comparisons are against baseline (* < 0.05, ** < 0.01, *** < 0.001). Colored asterisks refer to the corresponding colored curves. They show the comparison between BCVA value at a selected timepoint and the corresponding baseline value.

The mean BCVA was 0.43 ± 0.36 LogMAR at baseline, improving significantly to 0.37 ± 0.44 LogMAR two months after the latest DEXi (*p* = 0.02 vs. baseline). After FAi, the BCVA improved significantly at one (0.32 ± 0.45 LogMAR, *p* = 0.02), three (0.21 ± 0.27 LogMAR, *p* = 0.002), six (0.18 ± 0.23 LogMAR, *p* < 0.001) and twelve months (0.27 ± 0.35 LogMAR, *p* = 0.002) compared to the baseline values (Table 2 and Figure 1). Figure 1B presents the evolution of the BCVA depending on uveitis etiology, showing an overall improvement in all uveitis categories.

**Figure 2:**
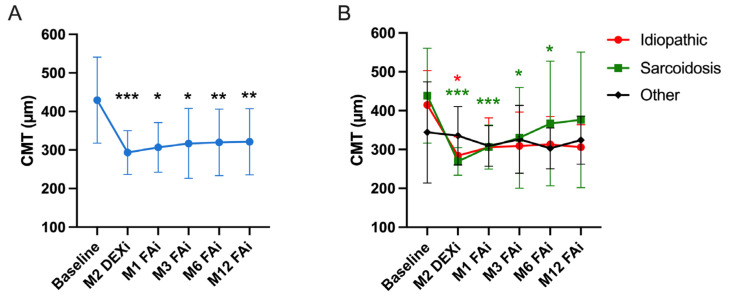
Evolution of the central macular thickness (CMT). (**A**) Whole cohort. (**B**) BCVA evolution according to uveitis etiology. “Baseline” refers to the pre-dexamethasone implant (DEXi) period. M: month. FAi: Fluocinolone acetonide implant. “Other” includes the following uveitis etiologies: Vogt–Koyanagi–Harada, autoimmune retinitis, HLA-B27+ and immune restauration syndrome. The graph represents the mean values with the corresponding standard deviation. Comparisons are against baseline (* < 0.05, ** < 0.01, *** < 0.001). Colored asterisks refer to the corresponding colored curves. They show the comparison between BCVA value at a selected timepoint and the corresponding baseline value.

The mean CMT was 429 ± 112 µm at baseline, improving significantly to 293 ± 56 µm two months after the latest DEXi (*p* = 0.0001 vs. baseline). After FAi, the CMT improved significantly at one (307 ± 65 µm, *p* = 0.015), three (317 ± 91 µm, *p* = 0.0025), six (320 ± 86 µm, *p* = 0.006) and twelve months (321 ± 85 µm, *p* = 0.004) compared to the baseline values (Table 2 and Figure 2). Figure 2B presents the evolution of the CMT depending on uveitis etiology, showing an overall improvement in all uveitis categories.

The evolution of the other inflammation parameters is displayed in Table 2. Briefly, the proportion of eyes showing signs of vitritis, optic nerve swelling and vasculitis decreased dramatically at the two-month visit following the latest DEXi, remaining stable (vasculitis) or decreasing further (vitritis, optic nerve swelling) at 1, 3, 6 and 12 months after FAi.

Similarly, the number of patients with macular edema decreased two months after the latest DEXi (from 100% to 34.6%, *p* < 0.0001), decreasing further after FAi (16.7%, 16.7%, 17.4% and 29.2% at 1, 3, 6, and 12 months, respectively; all *p* < 0.0001 vs. baseline). An either complete or incomplete (decrease >20% of the pre-injection CMT value without reaching complete dryness) resolution of macular edema was obtained in 73% of eyes two months after the latest DEXi, and in 91.6%, 87.4%, 86.9% and 79.1%, respectively, at 1, 3, 6 and 12 months after FAi (Table 3). 

The percentage of eyes with HRF was 53.8% (*n* = 14) at baseline, decreasing to 23.1% two months after the latest DEXi (*p* = 0.044). Interestingly, this proportion decreased further after FAi (4.1%, 8.3%, 8.7% and 20.8% at 1, 3, 6 and 12 months, respectively; all *p* < 0.05 vs. baseline). A similar trend was observed for SRD and DRIL (Table 3). 

### 3.3. Systemic Treatments

The proportion of patients receiving different types of systemic anti-inflammatory treatments is presented in Table 4. Briefly, the number of patients receiving systemic treatments decreased numerically after DEXi and FAi (13 patients (59.1%) at baseline, 11 patients (50%) after DEXi and 9 patients (45.0%) twelve months after FAi). The mean dose of CS decreased from 12.4 mg at baseline to 10.6 mg after the latest DEXi and to 8.3 mg twelve months after FAi, but this was not statistically significant.

### 3.4. Exploratory Predictive Factors of Functional and Anatomical Response to FAi

We explored the predictive factors for two outcomes: the best anatomical or functional result obtained during the follow-up and the anatomical or functional outcome obtained at the 12-month follow-up (Table 5). 

Briefly, regarding the anatomy, patients having received between 6 and 10 DEXi prior to FAi presented the highest decrease in their CMT at the 12-month follow-up (*p* = 0.03). In addition, a history of complete anatomical response after DEXi was associated with a higher likelihood of achieving a complete anatomical response at some point of the follow-up after FAi (*p* = 0.07). On the other hand, the presence of biomarkers of inflammation was not associated with a better nor worse anatomical response. However, patients displaying HRF at baseline seemed to decrease their CMT in a more consistent way compared to patients with no HRF, without statistical significance however (*p* > 0.05 for the two timepoints, Table 5). Patients receiving systemic corticosteroid treatment presented a better anatomical response compared to those with no associated therapy or those treated with immunosuppressive therapy, without being statistically significant.

Regarding the function, we noted a significative BCVA increase for uveitis etiologies other than idiopathic and sarcoidosis at the 12-month time point (*p* = 0.04). Considering the biomarkers of inflammation, patients with SRD presented a milder increase in their BCVA compared to those with no SRD at baseline (*p* ≤ 0.05 for the two timepoints, Table 5).

### 3.5. Predictive Factors of Rescue DEXi Injections

Six eyes (23%) received at least one rescue DEXi during the 12 months following FAi (Table 6). Among them, two had no concurrent systemic anti-inflammatory treatment, three were under the same regime of systemic anti-inflammatories after FAi, and the last had been undergoing a decrease in his steroid dosage after FAi, which could have played a role in indicating a rescue DEXi. The mean number of rescue DEXi injections was 0.35 ± 0.70 (0–2). More specifically, three eyes required two additional DEXi (two eyes were reinjected at 6 and 12 months, and the third eye was reinjected at 3 and 6 months) while the three remaining eyes required only one reinjection (at 3, 6 and 12 months, respectively). The only significative predictive factor for reinjection was the presence of DRIL at baseline (*p* < 0.001). The presence of HRF was not statistically associated with a higher likelihood of requiring rescue DEXi.

### 3.6. Adverse Events

**Figure 3:**
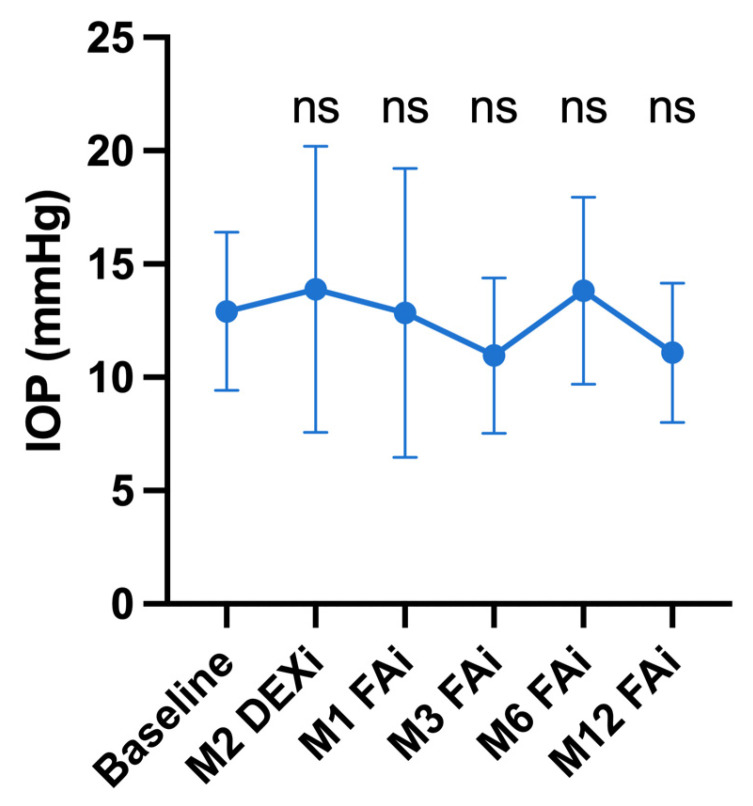
Evolution of the intra-ocular pressure (IOP). “Baseline” refers to the pre-dexamethasone implant (DEXi) period. M: month. FAi: Fluocinolone acetonide implant. The graph represents the mean values with the corresponding standard deviation. Comparisons are against baseline (ns: not statistically significant).

The mean pre-DEXi IOP was 12.9 ± 3.5 mmHg, remaining stable during the follow-up: 13.9 ± 6.3 (*p* = 0.42) two months after the latest DEXi, 12.9 ± 6.4 mmHg (*p* = 0.81) at one month, 11.0 ± 3.4 mmHg (*p* = 0.08) at 3 months, 13.8 ± 4.1 mmHg (*p* = 0.06) at 6 months and 11.1 ± 3.1 mmHg (*p* = 0.08) at 12 months following FAi (Table 7 and Figure 3). The proportion of eyes with ocular hypertension (i.e., IOP > 21 mmHg) was 0% (*n* = 0) before DEXi, 11.5% (*n* = 3, *p* = 0.62) two months after the latest DEXi, 7.7% (*n* = 2, *p* = 0.62), 0% (*n* = 0, *p* = 1), 3.8% (*n* = 1, *p* = 1) and 0% (*n* = 0, *p* = 1) at one, three, six and twelve months, respectively, after FAi.

The proportion of eyes with an IOP > 30 mmHg was 0% (*n* = 0) before DEXi, 3.8% (*n* = 1, *p* = 1) two months after the latest DEXi, 4.2% (*n* = 1, *p* = 0.5) one month following FAi and 0%, 0% and 0% (all *p* = 1) at three, six and twelve months, respectively.

Table 7 presents the evolution of anti-glaucoma treatments over time, showing a mean number of 0.35 ± 0.70 drops in the baseline period, changing to 0.50 ± 0.81 (*p* = 0.73) two months after the latest DEXi, 0.35 ± 0.75 (*p* = 0.98), 0.92 ± 1.30 (*p* = 0.05), 0.88 ± 1.3 (*p* = 0.08) and 0.88 ± 1.30 (*p* = 0.08) at one, three, six and twelve months following FAi. There was no additional filtering surgery after FAi as compared to the post-DEXi period. During the follow-up, one eye experienced hypotony (because of an untunneled scleral injection) requiring a sub-tenon injection of triamcinolone. No cases of endophthalmitis or retinal detachment were reported.

## 4. Discussion

The purpose of this work was to present real-life multicentric data from the French Uveitis Network, regarding the effectiveness of FAi (Iluvien^®^) in UME during a period spanning from September 2021 to October 2022.

Our cohort included 26 eyes with a mean follow-up of 11 months and a 12-month follow-up available for 24 eyes. The demographics of our patients included a slight majority of females (73%) with a wide range of ages (31 to 87 years) and a variety of uveitis etiologies, in keeping with tertiary care-unit enrollments. There was no case of UME associated with isolated anterior uveitis, and all eyes but two displayed at least one other sign of inflammation evident by ophthalmoscopy, in addition to the UME. Almost all patients were pseudophakic before FAi, which allowed us to investigate the functional outcomes independent of a cataract bias. A majority (59%) of patients were under systemic treatment with either corticosteroids, immunosuppressants or a combination of both, which matches the characteristics of previously published cohorts [22,23,24,25] but shows a certain degree of severity of the underlying disease. All patients had to have received at least two DEXi prior to the FAi for safety reasons. In fact, the PALADIN study showed that a previous corticosteroid challenge predicted an IOP elevation > 25 mmHg in 78% of diabetic macula edema cases treated with FAi [26]. We therefore chose to only include patients with a previous DEXi challenge and exclude those with a post-DEXi IOP increase not returning to normal (IOP < 21 mmHg) after either a mono or dual anti-glaucoma therapy. In this cohort, patients received FAi after a mean time of 5 months after the latest DEXi, which is longer than the 4- to 8-week interval that the French Uveitis Network currently recommends (data not published). In fact, it has been shown elsewhere that the best functional and anatomical results are reached faster in patients injected with FAi at the time of DEXi peak of action (i.e., around 4–8 weeks) [27,28].

Regarding the functional and anatomical results, our real-life study confirms the effectiveness of FAi that was previously shown in the pivotal randomized clinical trial [20], and also in multiple retrospective real-life studies that were however mainly monocentric, which may have induced a center-effect bias [22,24,25,29]. Our baseline BCVA was 0.43 LogMAR (Snellen equivalent 20/50), similar to Jaffe et al. [20]. On the other hand, our baseline CMT was slightly higher (429 microns), due to the fact that we only included patients with macular edema, while the rate of UME was only 56.5% in the pivotal study [20].

Over the 12-month period, the mean BCVA gain and CMT decrease compared to baseline were 0.16 LogMAR and 108 microns, respectively, which is comparable to other real-life monocentric studies [24]. More specifically, we showed that the BCVA gains (maximum gain of 0.6 LogMAR over the 12 month-follow-up) were statistically significant at all timepoints of the follow-up when compared to the baseline values. 

Similarly, the CMT decrease was statistically significant during the whole follow-up when compared to baseline. However, the CMT after FAi remained stable when compared to the post-DEXi value (Figure 2). The reason why the BCVA continued to improve after FAi despite an apparent CMT stability is probably due to the more sustained, less variable and longer-term improvement of the other inflammation parameters, including vitritis, papillitis and anterior chamber inflammation (Table 2). The impact of UME treatment on these parameters has only rarely been reported and deserves attention since it plays a major role in defining the BVCA outcomes. It is worth mentioning that only one eye lost vision during the follow-up, due to hypotony requiring a sub-tenon injection of triamcinolone.

Regarding the characteristics of macular edema, the majority (73.1%) of eyes displayed intraretinal fluid (IRF), in agreement with previous literature on UME [30]. Biomarkers of inflammation were present on OCT in a non-negligible proportion of patients, namely HRF in 53.8%, SRD in 38.5% and DRIL in 30.8% of cases, respectively.

Overall, we showed that FAi achieved the complete resolution of UME (complete dryness) in a higher proportion than DEXi (65.4% after DEXi vs. a maximum of 83.3% after FAi). Although DEXi was more efficient in resolving SRD (100% of cases), the proportion of eyes with no IRF increased further after FAi (maximum of 87.5% of eyes) than after DEXi (65.4%), which is of importance since it has been shown that IRF is associated with the worst long-term outcomes in case of chronicity [1]. The other inflammation biomarkers (HRF, DRIL) were also cleared more efficiently with FAi than with DEXi, suggesting the importance of a continuous and longer-term drug release [20,24,29].

Throughout the course of our study, the proportion of patients receiving different types of systemic anti-inflammatory treatments decreased in a non-statistically-significant way after FAi (31% at 12 months). The mean dose of CS also decreased during the follow-up, without reaching statistical significance, which can also be due to the limited follow-up duration. This is in line with other studies investigating the effects of FAi in uveitis and more specifically in UME, showing a decrease in the systemic treatment burden [31].

Ever since its FDA and EMA approval, only a few publications have investigated the predictive factors of treatment response in a real-world setting [29,32]. These predictive factors are important because they can allow the selection of patients that may benefit the most from treatment. We explored the predictive factors for responding to FAi by choosing three outcomes and two timepoints: BCVA gains, CMT decrease and complete UME resolution at the 12-month follow-up but also at the timepoint for which the best result has been observed (Table 5). Although not statistically significant, it seemed that UME eyes in the context of idiopathic uveitis and intermediate uveitis were the most likely to improve their CMT compared to the other uveitis subtypes. To our knowledge, this has never been reported thus far. Although not statistically significant, a history of complete anatomical response after DEXi was associated with the likeliest complete anatomical response after FAi. While this has been reported in the context of diabetic macular edema [33], to our knowledge a prechallenge with DEXi has never been investigated as a predictive factor of the response to FAi in UME, and it may be of interest to help with the screening of patients. On the other hand, the presence of biomarkers of inflammation was not associated with a better nor worse anatomical response, which may be due to the relatively small sample that was included in this cohort.

While decreasing the systemic treatment burden, FAi also decreased the local treatment burden, with an average of 1.5 DEXi injections per year during the pre-FAi period, decreasing to an average of 0.35 DEXi injections per year after FAi. Regarding the predictive factors of rescue DEXi injections and due to the limited size of the sample, we only performed an exploratory univariate analysis. We found that the presence of DRIL at baseline (*p* < 0.001) was the only pejorative variable, which has never been reported thus far in the context of uveitis. However, DRIL has been reported to be associated with low BCVA figures in diabetes and uveitis patients with or without macular edema [34], and it has also been shown to be associated with lesser CMT improvements in diabetic macular edema compared to eyes showing no DRIL [35]. The presence of HRF was seen in 83.3% of eyes requiring rescue DEXi injections compared to 10.0% in the group requiring no rescue. Although not statistically significant, this trend suggests that HRF may be associated with a higher requirement of further DEXi injections.

Together, these data may identify the subgroups of patients who may be more suitable for treatment with FAi. Further studies with larger samples are nonetheless warranted to confirm the trends that we report in this work.

Regarding the safety, 25 of 26 eyes were pseudophakic before the FAi injection and the remaining eye underwent cataract surgery one month after FAi.

The mean IOP was overall stable throughout the follow-up (i.e., changes from baseline were not significant) in keeping with the safety data published thus far [20,29]. In addition, while there was a slight but non-statistically significant increase in the number of anti-glaucoma drops that were used from Month 3 to Month 12 in comparison to baseline and the immediate post-DEXi period, no patient underwent filtering surgery. This confirms the safety results of the pivotal clinical trial that found less risk of glaucoma surgery in FAi implanted eyes vs. simulated injection over a 36-month follow-up [20].

There are limitations to this study that need to be acknowledged. Firstly, the relatively small number of included eyes may have affected the statistical power of our analyses, especially those directed at identifying the predictive factors of response to treatments. Secondly, the follow-up duration was limited to 12 months, which restricts our long-term conclusions. However, it needs to be reminded that the FAi implant did not receive market authorization until 2021 in France (for the indication of uveitis), which explains the scarcity of long-term data. Lastly, the retrospective nature of this survey, and the lack of control group or parallel comparison with other intravitreal corticosteroids drugs is another limitation that needs to be mentioned. In the meantime, one of the strengths of this study was its multicentric design that limits the center-effect bias.

Overall, this study demonstrates in a multicentric and real-world fashion the effectiveness of the fluocinolone acetonide implant in treating uveitic macular edema, improving visual acuity, reducing the central retinal thickness and decreasing the recourse to systemic and local anti-inflammatory treatments. The safety profile was acceptable, with no reported serious adverse events in keeping with the literature. 

## Figures and Tables

**Table 1:** Characteristics of patients and eyes at baseline (before FAi).

*n* = 26 Eyes (22 Patients)	*n* (%) or Mean ± SD (Range)
Gender (females), *n* (%)	16 (73.1%)
Mean age (years)	60.4 ± 15.8 (31–87)
Mean follow-up duration (months)	11.4 ± 2.0 (3–12)
Uveitis type, *n* (%)	
Anterior Intermediate Posterior Panuveitis	0 (0%) 3 (11.5%) 9 (34.6%) 14 (53.8%)
Etiology of uveitis, *n* (%)	
Idiopathic Sarcoidosis Vogt–Koyanagi–Harada Autoimmune HLA-B27+ Immune restauration syndrome	15 (57.7%) 5 (19.2%) 2 (7.7%) 2 (7.7%) 1 (3.8%) 1 (3.8%)
Baseline signs of inflammation, *n* (%)	
Anterior chamber cells Vitreous haze Vasculitis Optic nerve swelling Macular edema Serous retinal detachment (SRD) Cystoid spaces Hyper-reflective foci (HRF) Disorganization of the inner retinal layers (DRIL)	11 (42.3%) 17 (65.4%) 10 (38.5%) 10 (38.5%) 26 (100%) 10 (38.5%) 19 (73.1%) 14 (53.8%) 8 (30.8%)
Lens status before FAi, *n* (%)	
Pseudophakic	25 (96.2%)
Number of antiglaucoma drops before FAi, *n* (%)	
0 1 2 3 4	20 (76.9%) 3 (11.5%) 3 (11.5%) 0 (0%) 0 (0%)
Number of DEXi per eye before FAi, *n* (%)	
Not mentioned 2–5 5–10 10–20 >20 Mean number (per eye)	2 (7.7%) 7 (26.9%) 11 (42.3%) 5 (19.2%) 1 (3.8%) 8.5 ± 6.1 (2–33)
Use of systemic treatments before FAi, *n* (%) Corticosteroids only Immunosuppressive treatment only Corticosteroids + immunosuppressive treatment	13 (59.1%) 3 (13.6%) 5 (22.7%) 5 (22.7%)

*n* = number, % = percentage, SD = standard deviation, FAi = fluocinolone acetonide implant, HLA = human-leukocyte antigen, DEXi = dexamethasone implant.

**Table 2:** Evolution of key characteristics based on treatments.

	Baseline	After Latest DEXi	After FAi			
		M2	M1	M3	M6	M12
	*n* (%) or Mean ± SD (Range)					
Eyes	26	26	24	24	23	24
BCVA (LogMAR), Mean ± SD (range)	0.43 ± 0.36 (1.40–0)	0.37 ± 0.44 (1.40–0)	0.32 ± 0.45 (1.70–0.08)	0.21 ± 0.27 (1.10–0.08)	0.18 ± 0.23 (1–0.08)	0.27 ± 0.35 (1.30–0.08)
Central macular thickness (µm), Mean ± SD (range)	429 ± 112 (275–617)	293 ± 56 (216–439)	307 ± 65 (226–483)	317 ± 91 (218–544)	320 ± 86 (223–591)	321 ± 85 (217–626)
Intraocular pressure (mmHg), Mean ± SD (range)	12.9 ± 3.5 (8–21)	13.9 ± 6.3 (5–25)	12.9 ± 6.4 (5–33)	11.0 ± 3.4 (5–21)	13.8 ± 4.1 (7–26)	11.1 ± 3.1 (7–19)
Glaucoma treatment, *n* (%)						
None Monotherapy Dual therapy Triple therapy Quadruple therapy Filtering surgery	20 (71.4%) 3 (11.5%) 3 (11.5%) 0 (0%) 0 (0%) 0 (0%)	15 (57.7%) 4 (15.4%) 5 (19.2%) 0 (0%) 0 (0%) 2 (7.7%)	15 (62.5%) 3 (12.5%) 6 (25.0%) 0 (0%) 0 (0%) 0 (0%)	13 (54.2%) 2 (8.3%) 7 (29.2%) 0 (0%) 2 (8.3%) 0 (0%)	12 (52.2%) 2 (8.7%) 7 (30.4%) 0 (0%) 2 (8.7%) 0 (0%)	13 (54.2%) 2 (8.3%) 7 (29.2%) 0 (0%) 2 (8.3%) 0 (0%)
Anterior chamber cells (+) ^a^, Mean ± SD (range)	0.8 ± 1.2 (0–3)	0.0 ± 0.0 (0–0)	0.0 ± 0.0 (0–0)	0.08 ± 0.3 (0–1)	0.0 ± 0.0 (0–0)	0.2 ± 0.6 (0–2)
Anterior chamber cells, N (%)	11 (42.3%)	1 (3.8%)	0 (0%)	1 (4.2%)	0 (0%)	0 (0%)
Vitreous Haze (+) ^a^, Mean ± SD (range)	1.11 ± 0.80 (0–3)	0.14 ± 0.40 (0–1)	0.11 ± 0.20 (0–0.5)	0.04 ± 0.10 (0–0.5)	0.0 ± 0.0 (0–0)	0.0 ± 0.0 (0–0)
Vitritis, *n* (%)	17 (65.4%)	4 (15.4%)	2 (8.3%)	1 (4.2%)	0 (0%)	0 (0%)
Optic nerve swelling, *n* (%)	10 (38.5%)	4 (15.4%)	1 (4.2%)	0 (0%)	0 (0%)	0 (0%)
Retinal vasculitis, *n* (%)	10 (38.5%)	1 (3.8%)	1 (4.2%)	1 (4.2%)	0 (0%)	0 (0%)
Dexamethasone rescue injection, *n* (%)	/	/	0 (0%)	2 (8.3%)	4 (17.4%)	3 (12.5%)
FAi reinjection, *n* (%)	/	/	0 (0%)	0 (0%)	0 (0%)	1 (4.2%)

*n* = number, % = percentage, SD = standard deviation, FAi = fluocinolone acetonide implant, DEXi = dexamethasone implant, BCVA = best corrected visual acuity, ^a^—grade according to the standardization of uveitis nomenclature criteria [21].

**Table 3:** Evolutive characteristics of macular edema.

	Baseline	After Latest DEXi	After FAi			
		M2	M1	M3	M6	M12
	*n* (%) or Mean ± SD (Range)					
Eyes	26	26	24	24	23	24
Presence of macular edema, *n* (%) HRF SRD Cystoid spaces DRIL	26 (100%) 14 (53.8%) 10 (38.5%) 19 (73.1%) 8 (30.8%)	9 (34.6%) 6 (23.1%) 0 (0%) 9 (34.6%) 3 (11.5%)	4 (16.7%) 1 (4.1%) 1 (4.1%) 3 (12.5%) 0 (0%)	4 (16.7%) 2 (8.3%) 1 (4.1%) 3 (12.5%) 2 (8.3%)	4 (17.4%) 2 (8.7%) 1 (4.3%) 4 (17.4%) 2 (8.7%)	7 (29.2%) 5 (20.8%) 1 (4.1%) 3 (12.5%) 4 (16.6%)
Complete resolution of UME, *n* (%)	/	17 (65.4%)	20 (83.3%)	20 (83.3%)	19 (82.6%)	17 (70.8%)
Incomplete resolution of UME, *n* (%)	/	2 (7.6%)	2 (8.3%)	1 (4.1%)	1 (4.3%)	2 (8.3%)
Absence of CMT decrease, *n* (%)		3 (11.5%)	0 (0%)	2 (8.3%)	2 (8.7%)	4 (16.6%)

*n* = number, % = percentage, SD = standard deviation, M = month, DEXi = dexamethasone implant, FAi = fluocinolone acetonide implant, HRF = hyperreflective foci, SRD = subretinal detachment, DRIL = disorganization of the inner retinal layers, UME = uveitic macular edema, CMT = central macular thickness. Incomplete resolution of UME refers to a CMT decrease >20% of its baseline value without reaching complete dryness.

**Table 4:** Associated systemic treatments.

	Baseline	After Latest DEXi	After FAi			
			M1	M3	M6	M12
	*n* (%) or Mean ± SD (Range)					
Patients	22	22	20	20	19	20
Associated systemic treatment, *n* (%) Corticosteroids (CS) only Immunosuppressive (IS) therapy only CS + IS	13 (59.1%) 3 (13.6%) 5 (22.7%) 5 (22.7%)	11 (50.0%) 3 (13.6%) 6 (27.2%) 2 (9.1%)	10 (50.0%) 5 (25.0%) 4 (20.0%) 1 (5.0%)	10 (50.0%) 5 (25.0%) 4 (20.0%) 1 (5.0%)	10 (52.6%) 5 (26.3%) 4 (21.1%) 1 (5.3%)	9 (45.0%) 2 (10.0%) 4 (20.0%) 3 (15.0%)
CS dose (milligrams) among treated, Mean ± SD (range)	12.4 ± 5.6 (5–20)	10.6 ± 5.3 (5–20)	9.5 ± 5.4 (4–20)	8.9 ± 5.0 (4–20)	8.8 ± 5.1 (3–20)	8.3 ± 6.2 (3–20)
CS dose, *p*-value vs. baseline	Ref	0.50	0.19	0.19	0.19	0.37
Anti TNF, *n* (%)	4 (18.2%)	5 (22.7%)	3 (15.0%)	3 (15.0%)	3 (15.8%)	5 (25.0%)
Interferon, *n* (%)	1 (4.5%)	1 (4.5%)	1 (5.0%)	1 (5.0%)	1 (5.3%)	1 (5.0%)
Methotrexate, *n* (%)	3 (13.6%)	3 (13.6%)	3 (15.0%)	3 (15.0%)	3 (15.8%)	3 (15.0%)
Hydroxychloroquine, *n* (%)	1 (4.5%)	1 (4.5%)	1 (5.0%)	1 (5.0%)	1 (5.3%)	1 (5.0%)
Tocilizumab, *n* (%)	2 (9.1%)	0 (0%)	0 (0%)	0 (0%)	0 (0%)	0 (0%)

*n* = number, % = percentage, SD = standard deviation, M = month, DEXi = dexamethasone implant, FAi = fluocinolone acetonide implant, CS = corticosteroids, IS = immunosuppressive, TNF = tumor necrosis factor.

**Table 5:** Baseline predictive factors of functional and anatomical response to FAi.

	Best Result during Follow-up						12 Month Follow-up					
	Mean BCVA Gain (LogMAR)	*p*	Mean CMT Decrease (µm)	*p*	Complete Anatomical Response *n* (%)	*p*	Mean BCVA Gain (LogMAR)	*p*	Mean CMT Decrease (µm)	*p*	Complete Anatomical Response *n* (%)	*p*
Age												
-<60 years ->60 years	0.27 0.16	Ref 0.47	110 178	Ref 0.08	12 (100%) 12 (86%)	Ref 0.48	0.17 0.20	Ref 0.25	61 154	Ref 0.20	10 (83.3%) 7 (58.3%)	Ref 0.37
Gender												
-Female -Male	0.27 0.16	Ref 0.48	110 178	Ref 0.25	18 (94.7%) 6 (85.7%)	Ref 0.47	0.23 0.07	Ref 0.23	77 191	Ref 0.14	12 (66.7%) 5 (83.3%)	Ref 0.63
Etiology of uveitis												
-Idiopathic -Sarcoidosis -Others	0.20 0.17 0.35	Ref 0.66 0.65	143 140 96	Ref 0.88 0.40	15 (100%) 4 (80.0%) 5 (83.3%)	Ref 0.25 0.29	0.13 0.17 0.33	Ref 0.83 0.04	128 92 81	Ref 0.86 0.34	12 (80.0%) 3 (75.0%) 2 (40%)	Ref 1 0.13
Type of uveitis												
-Posterior -Intermediate -Panuveitis	0.36 0.15 0.17	Ref 0.71 0.24	11 115 129	Ref 0.48 0.90	8 (88.9%) 3 (100%) 13 (92.9%)	Ref 1 1	0.31 0.07 0.09	Ref 0.60 0.26	100 223 96	Ref 0.33 0.74	6 (66.7%) 2 (100%) 9 (69.2%)	Ref 1 1
Number of previous DEXi												
-1–5 -6–10 ->10	0.22 0.24 0.32	Ref 0.81 0.55	53 193 58	Ref 0.07 1	5 (83.3%) 10 (90.9%) 6 (100%)	Ref 1 1	0.22 0.16 0.24	Ref 1 0.97	−13 188 46	Ref 0.03 0.69	3 (60%) 6 (60%) 5 (83.3)	Ref 1 0.42
Complete anatomic response to DEXi												
-No -Yes	0.34 0.19	Ref 0.51	90 89	Ref 0.82	4 (66.7%) 15 (100%)	Ref 0.07	0.36 0.11	Ref 0.45	24 71	Ref 0.55	1 (25.0%) 11 (73.3%)	Ref 0.12
Partial anatomic response to DEXi												
-No -Yes	0.44 0.16	Ref 0.53	102 65	Ref 1	2 (50.0%) 2 (100%)	Ref 0.47	0.45 0.08	Ref 0.80	16 49	Ref 1	0 (0%) 1 (100%)	Ref 0.25
Vitritis grade ^a^:												
-<1+ -≥1+	0.28 0.22	Ref 0.38	145 119	Ref 0.53	13 (100.0%) 11 (84.6%)	Ref 0.48	0.21 0.17	Ref 0.61	122 98	Ref 0.54	9 (69.2%) 8 (72.7%)	Ref 1
Posterior inflammation other than UME												
-No -Yes	0.28 0.23	Ref 0.51	172 115	Ref 0.36	9 (100.0%) 15 (88.2%)	Ref 0.53	0.23 0.17	Ref 0.41	147 95	Ref 0.48	6 (66.7%) 11 (73.3%)	Ref 1
Associated systemic treatment at the initial visit:												
-None -CS only -IS rx only -CS + IS rx	0.40 0.10 0.18 0.16	Ref 0.14 0.71 0.35	145 172 107 121	Ref 1 0.67 0.67	5 (71.4%) 3 (100%) 5 (100%) 7 (100%)	Ref 1 0.47 1	0.38 0.09 0.14 0.06	Ref 0.31 0.86 0.52	83 171 101 101	Ref 0.55 0.91 0.91	4 (57.1%) 3 (100%) 3 (75.0%) 5 (71.4%)	Ref 0.29 1 1
Presence of biomarkers HRF												
-None -Presence SRD -None -Presence DRIL -None -Presence	0.11 0.33 0.35 0.07 0.16 0.32	Ref 0.17 Ref 0.03 Ref 0.15	79 163 121 143 152 160	Ref 0.09 Ref 0.75 Ref 0.95	11 (91.7%) 13 (92.3%) 15 (93.7%) 9 (90.0%) 13 (92.9%) 7 (87.5%)	Ref 1 Ref 1 Ref 1	0.04 0.28 0.28 0.01 0.12 0.25	Ref 0.26 Ref 0.05 Ref 0.16	43 151 100 126 139 138	Ref 0.07 Ref 0.87 Ref 0.92	8 (72.7%) 9 (69.2%) 10 (62.5%) 7 (87.5%) 10 (83.3%) 5 (62.5%)	Ref 1 Ref 0.35 Ref 0.35

*n* = number, % = percentage, DEXi = dexamethasone implant, FAi = fluocinolone acetonide implant, HRF = hyperreflective foci, SRD = subretinal detachment, DRIL = disorganization of the inner retinal layers, UME = uveitic macular edema, CMT = central macular thickness. Partial anatomic response refers to a CMT decrease >20% of its baseline value, BCVA = Best-corrected visual acuity, CS = corticosteroids, IS = immunosuppressive, Rx = treatment. **^a^**–grade according to the standardization of uveitis nomenclature criteria [21]. Ref= taken as reference for analysis.

**Table 6:** Predictive factors of rescue DEXi injections.

	Eyes without Additional DEXi *n* = 20	Eyes with at Least One Rescue DEXi *n* = 6	*p*-Value
Age			
-<60 years ->60 years	10 (83.3%) 10 (71.4%)	2 (16.7%) 4 (28.6%)	Ref 0.65
Gender			
-Female -Male	16 (84.2%) 4 (57.1%)	3 (15.8%) 3 (42.9%)	Ref 0.29
Etiology of uveitis			
-Idiopathic -Sarcoidosis -Others	12 (80.0%) 5 (100%) 3 (50.0%)	3 (20.0%) 0 (0%) 3 (50.0%)	Ref 0.54 0.29
Type of uveitis			
-Posterior -Intermediate -Panuveitis	5 (55.6%) 3 (100%) 12 (85.7%)	4 (44.4%) 0 (0%) 2 (14.3%)	Ref 0.49 0.16
Number of previous DEXi			
-1–5 -6–10 ->10 -No data	6 (85.7%) 7 (63.4%) 5 (83.3%) 2 (100%)	1 (16.7%) 4 (36.4%) 1 (16.7%) 0 (0%)	Ref 0.60 1 -
Complete anatomic response to DEXi			
-No -Yes -No data	5 (83.3%) 10 (66.7%) 5 (100%)	1 (16.7%) 5 (33.3%) 0 (0%)	Ref 0.62 -
Partial anatomic response to DEXi			
-No * -Yes **	3 (75.0%) 2 (100%)	1 (25.0%) 0 (0.0%)	Ref 1
Associated systemic treatment at Baseline			
-None -Corticosteroids only -Immunosuppressive rx only -Corticosteroids + immunosuppressive rx -No data	5 (55.6%) 3 (100%) 6 (100%) 4 (66.7%) 2 (100%)	4 (44.4%) 0 (0%) 0 (0%) 2 (33.3%) 0 (0%)	Ref 0.49 0.10 0.49 -
Presence of biomarkers at baseline			
HRF -None -Presence SDR -None -Presence DRIL -None -Presence -No data	11 (91.7%) 9 (64.3%) 11 (68.7%) 9 (90.0%) 14 (100%) 2 (25.0%) 4 (100%)	1 (8.33%) 5 (35.7%) 5 (31.3%) 1 (10.0%) 0 (0%) 6 (75.0%) 0 (0%)	Ref 0.17 Ref 0.35 Ref <0.001 -

*n* = number, % = percentage, DEXi = dexamethasone implant, HRF = hyperreflective foci, SRD = subretinal detachment, DRIL = disorganization of the inner retinal layers, Rx = treatment, Partial anatomic response refers to a decrease in CMT > 20% of its baseline value. * *n* = 4, ** *n* = 2. Ref= taken as reference for analysis.

**Table 7:** Evolution of intraocular pressure parameters.

	Baseline	After Latest DEXi	After FAi			
		M2	M1	M3	M6	M12
	*n* (%) or Mean ± SD (Range)					
Eyes	26	26	24	24	23	24
Intraocular pressure (mmHg), Mean ± SD (range)	12.9 ± 3.5 (8–21)	13.9 ± 6.3 (5–25)	12.9 ± 6.4 (5–33)	11.0 ± 3.4 (5–21)	13.8 ± 4.1 (7–26)	11.1 ± 3.1 (7–19)
*p*-value vs. baseline	Ref	0.42	0.81	0.08	0.06	0.08
Eyes with IOP > 21 mmHg, *n* (%)	0 (0%)	3 (11.5%)	2 (8.3%)	0 (0%)	1 (4.3%)	0 (0%)
*p*-value vs. baseline	Ref	0.62	0.62	1	1	1
Eyes with IOP > 30 mmHg, *n* (%)	0 (0%)	1 (3.8%)	1 (4.2%)	0 (0%)	0 (0%)	0 (0%)
*p*-value vs. baseline	Ref	1	0.50	1	1	1
Glaucoma treatment, *n* (%)						
No treatment Monotherapy Dual therapy Triple therapy Quadruple therapy Filtering surgery	20 (71.4%) 3 (11.5%) 3 (11.5%) 0 (0%) 0 (0%) 0 (0%)	15 (57.7%) 4 (15.4%) 5 (19.2%) 0 (0%) 0 (0%) 2 (7.7%)	15 (62.5%) 3 (12.5%) 6 (25.0%) 0 (0%) 0 (0%) 0 (0%)	13 (54.2%) 2 (8.3%) 7 (29.2%) 0 (0%) 2 (8.3%) 0 (0%)	12 (52.2%) 2 (8.7%) 7 (30.4%) 0 (0%) 2 (8.7%) 0 (0%)	13 (54.2%) 2 (8.3%) 7 (29.2%) 0 (0%) 2 (8.3%) 0 (0%)
*n* of anti-glaucoma drops, Mean ± SD (range)	0.35 ± 0.70	0.50 ± 0.81	0.35 ± 0.75	0.92 ± 1.30	0.88 ± 1.30	0.88 ± 1.30
*p*-value vs. baseline	Ref	0.73	0.98	0.05	0.08	0.08

*n* = number, % = percentage, SD = standard deviation, DEXi = dexamethasone implant, FAi = fluocinolone acetonide implant, M = month, IOP = intraocular pressure.

## Data Availability

Data are contained within the article.

## Supplementary Materials

The following supporting information can be downloaded at: https://www.mdpi.com/article/10.3390/jpm14030245/s1, Figure S1: Optical coherence tomography scans of the right macula of an idiopathic uveitis patient. (A) At baseline. (B) Two months after the 4th dexamethasone implant. (C) Twelve months after the fluocinolone acetonide implant.
