# Supplementary material for: Efficacity and Safety of the Fluocinolone Acetonide Implant in Uveitic Macular Edema: A Real-Life Study from the French Uveitis Network
Source: J Pers Med. 2024 Feb 24;14(3):245. doi: 10.3390/jpm14030245 (PMC10971732; doi:10.3390/jpm14030245)
Supplement: Supplementary file 1 [file jpm-14-00245-s001.zip › jpm-2864529-supplementary.pdf]

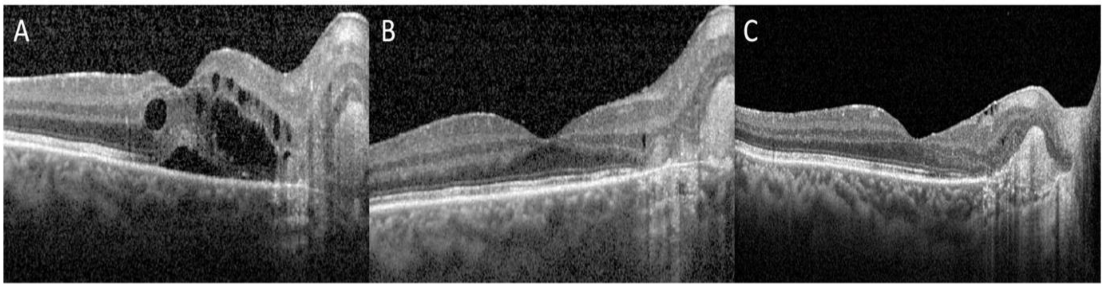

Supplemental Figure S1: Optical coherence tomography scans of the right macula of an idiopathic uveitis patient. (**A**) At baseline. (**B**) Two months after the 4th dexamethasone implant. (**C**) Twelve months after the fluocinolone acetonide implant.
